# High muscular fitness has a powerful protective cardiometabolic effect in adults: influence of weight status

**DOI:** 10.1186/s12889-016-3678-5

**Published:** 2016-09-23

**Authors:** Robinson Ramírez-Vélez, Jorge E. Correa-Bautista, Felipe Lobelo, Mikel Izquierdo, Alicia Alonso-Martínez, Fernando Rodríguez-Rodríguez, Carlos Cristi-Montero

**Affiliations:** 1 Center for Studies on Measurement of Physical Activity, School of Medicine and Health Sciences, Universidad del Rosario, Bogotá, D.C Colombia; 2 GICAEDS Group, Faculty of Physical Culture, Sport and Recreation, Universidad Santo Tomás, Bogotá, D.C Colombia; 3 Hubert Department of Global Health, Rollins School of Public Health, Emory University, Atlanta, GA USA; 4 Department of Health Sciences, Public University of Navarra (Navarra) SPAIN, Campus of Tudela, Av. de Tarazona s/n. 31500, Tudela, Navarra Spain; 5 IRyS Group. School of Physical Education. Pontificia Universidad Católica de Valparaíso, Valparaiso, Chile

**Keywords:** Muscle strength, Cardiovascular diseases, Cholesterol, Cardiometabolic risk, Exercise

## Abstract

**Background:**

Low levels of muscular fitness (MF) are recognized as an important marker of nutritional status and a predictor of metabolic complications, cardiovascular disease and death, however, the relationship between MF, body mass index (BMI) and the subsequent cardiometabolic protective effects has been less studied among Latin American populations. This study identified an association between MF and the cardiometabolic risk score index (CMRSI) and the lipid-metabolic cardiovascular risk index (LMCRI) in a wide sample of university students grouped according to their BMI.

**Methods:**

Six thousand ninety five healthy males (29.6 ± 11.7 year-old) participated in the study. Absolute strength was measured using a T.K.K. analogue dynamometer (handgrip), and the participant’s strength was then calculated relative to their body mass (MF/BM). The LMCRI was derived from the levels of triglycerides, low-density lipoprotein cholesterol (LDL-c), high-density lipoprotein cholesterol (HDL-c), and glucose levels in a blood sample. The CMRSI was calculated by summing the standardized residuals (z-score) for waist circumference, total cholesterol, LDL-c, triglycerides, HDL-c, and median blood pressure. Subjects were divided into six subgroups according to BMI (normal vs. overweight/obese) and MF/BM tertiles (unfit, average, fit).

**Results:**

The group of participants with low and moderate levels of MF/BM showed higher CMRSI values independent of BMI (*P* < 0.001). The group with normal BMI and high MF/BM had the highest levels of cardiometabolic protection. All overweight/obese BMI groups had significantly higher LMCRI values independent of the level of MF/BM (*P* < 0.001).

**Conclusions:**

Participants with high MF/BM showed reduced cardiometabolic risk, which increased significantly when they were within normal parameters.

## Background

Low level of MF is known to be an important marker of nutritional status [1, 2], metabolic syndrome [3], cancer [2], cardiovascular disease [3, 4] and all-cause mortality [5, 6], and these associations are much stronger in men than in women [1, 5].

Evidence is also accumulating that MF may be inversely associated with cardiometabolic risk factors from an early age [4, 6], and that lower MF in young adulthood can predict cardiovascular disease and mortality in adulthood [6] independent of body mass index and cardiorespiratory fitness [2, 6]. There are also indications that lower-body MF is inversely related to abdominal adiposity and that a composite strength score (using handgrip, standing broad jump, and a muscle endurance indicator) is related to a positive lipid profile and improved glucose levels [4].

Most evidence to date about the association between MF levels and health has been gathered using Caucasian cohorts [3, 7]. Some differences have also been found in muscular fitness levels according to the type of population studied [8], which may influence its association with health [9]. An important cross-sectional study found no significant differences when comparing the handgrip strength of Caucasian, Hispanic and African-American males (values adjusted by age, height, body mass, etc.), however, when relating these values to lean arm mass, the Caucasian population was found to have higher strength levels than the other two groups [8]. These results indicate the importance of studying different populations while taking into account their individual characteristics [9].

Clinical examinations and handgrip measurements are described in detail by Ruiz et al. [2] Artero et al. [3] and Cadenas-Sanchez et al. [10], respectively. The term ‘MF’ has been used to represent muscular strength, local muscular endurance and muscular power [11, 12]. Muscular strength is understood as the ability to generate force with a muscle or group of muscles, and local muscular endurance is the ability to perform repeated contractions with a muscle or group of muscles under sub-maximal load. Muscular power refers to the rate at which muscles can perform work.

Typically, handgrip strength can be measured using relatively inexpensive, portable and easy-to-use dynamometers, which are considered a reliable and valid method of strength assessment [13, 14]. Collective MF can be assessed using various strength performance tests such as handgrip strength, explosive lower-limb power (jumps), and muscular endurance (sit-ups) [10]. From a public health perspective, the inclusion of MF in health surveillance systems is therefore clearly justifiable and could be an ideal tool for monitoring youth fitness and identifying those with poor strength. This study identified the association between MF and the cardiometabolic risk scores in a wide sample of university students grouped according to their BMI.

## Methods

### Study population

We conducted the cross-sectional component of the FUPRECOL study (Association between Muscular Strength and Metabolic Risk Factors in Colombia) in Bogota, Colombia during the 2013–2014 college year. The sample comprised 6,095 apparently healthy male adult volunteers aged 18–40 years (mean, 29.6 ± 11.7 years) of low to middle socioeconomic status (SES: 1–4 on a scale of 1–6 defined by the Colombian government) and enrolled in public or private university in the capital district of Bogota, Cundinamarca Department in the Andean region. This region is located at approximately 4°35′56″N 74°04′51″W and at an elevation of approximately 2625 m (range 2500–3250) above sea level. Bogota is considered an urban area, with approximately 7,862,277 inhabitants. Inclusion criteria were: i) no movement restriction in the upper extremities; ii) no self-reported history of inflammatory joint disease, neurological disorder or injury to the upper extremity; and iii) not an athlete participating at an elite level. Volunteers were not compensated for their participation. Subjects with a medical or clinical diagnosis of a major systemic disease (including malignant conditions such as cancer), type 1 or 2 diabetes mellitus, high blood pressure, hypothyroidism/hyperthyroidism, a history of drug or alcohol abuse, regular use of multivitamins or inflammatory (trauma, contusions) or infectious conditions were also excluded from the study.

### Measures

#### Anthropometric

All measurements were obtained at the same time of the day (between 7:00–9:30 AM). Anthropometric variables were measured by a Level 2 expert certified by the International Society for the Advancement of Kinanthropometry (ISAK) in accordance with ISAK guidelines [15]. Body weight was measured with the subjects wearing only underwear and no shoes, using electronic scales (Tanita® BC544, Tokyo, Japan) with a low technical error of measurement (TEM = 0.510 %). Height was measured using a mechanical stadiometer platform (Seca® 274, Hamburg, Germany; TEM = 0.019 %). BMI was calculated as body weight in kilograms divided by the square of the height in meters. Normal BMI was defined as 18.5–24.9 and overweight was defined as ≥ 25.0 kg/cm^2^ according to the World Health Organization’s standards [16]. Waist circumference (WC) was measured at the midpoint between the last rib and the iliac crest using a tape measure (Ohaus® 8004-MA, New Jersey, USA; TEM = 0.086 %). A detailed questionnaire was used to collect information on diseases diagnosed by physicians.

### Muscular fitness

MF was measured using a standard adjustable handle Takei Digital Grip Strength Dynamometer Model T.K.K.540® (Takei Scientific Instruments Co., Ltd, Niigata, Japan). The participants were given a brief demonstration and verbal instructions for the test, and if necessary, the dynamometer was adjusted to hand size according to predetermined protocols [10]. Grip strength was measured with the subject in a standing position with the shoulder adducted and neutrally rotated, and arms parallel but not in contact with the body. Participants were asked to squeeze the handle for a maximum of 3–5 s; no verbal encouragement was given during the test. Two trials were allowed for each limb and the average score was recorded as the peak grip strength (kg). The grip strength values presented here thus combine the results of left- and right-handed subjects, without consideration of hand dominance. All study personnel were trained in testing and calibration procedures, and a calibration log was maintained. The systematic error when the grip strength assessments were performed twice was 0.508 (95 % CI = −3.078 to 4.094 %; n = 207). To account for differences in body size, MF divided by body mass (MF/BM) was used in the subsequent analysis (tertiles: unfit, average, fit).

### Clinical and biomarkers

After fasting for 12 h, blood samples were obtained from an antecubital vein at 6:30 AM–7: 00 AM, and prior to the fitness tests. Participants were asked to not participate in any prolonged exercise for the 24 h prior to testing. After a light breakfast (approximately 450 kcal), blood pressure was measured twice from the left hand via an Omron M6 Comfort (Omron® Healthcare Europe B.V., Hoofddorp, the Netherlands) while the participants were sitting still. The blood pressure monitor cuff was placed two to three finger widths above the bend of the arm and a 2-min pause was allowed between the first and the second measurements. The mean blood pressure (MBP) was calculated using the following formula: MBP = (systolic blood pressure + (2 × diastolic blood pressure)) / 3.

The glucose and lipid profiles were assessed using a routine colorimetric method (BTS-303 Biosystem photometric, Barcelona, Spain). Low-density lipoprotein cholesterol (LDL-c) was calculated using the Friedewald formula only if triglyceride levels were below 400 mg/dL [17].

### Statistical analysis

Analysis of the variables and measurements was performed using SPSS V. 21.0 software for Windows (SPSS, Chicago, Illinois, USA), and the significance level was set at *P* < 0.05. A Kolmogorov-Smirnov test was used to assess normality for all variables. Means and SD for anthropometric variables, MF, clinical and biomarker measurements were calculated. An ANOVA test was used to examine any significant difference by BMI group. We calculated a continuous composite cardiometabolic risk factor score index (CMRSI) by summing standardized residuals (Z-scores) for waist circumference, total cholesterol, LDL-c, triglycerides, HDL-c, and MBP. These variables are used as the criteria for metabolic syndrome in adults (18). A lipid-metabolic cardiovascular risk index (LMCRI) was derived from the levels of triglycerides, LDL-c, HDL-c, and glucose [18]. For each of these variables, a Z-score was computed as the number of standard deviation (SD) units from the sample mean after normalization of the variables, i.e.: Z-score = ((value – sample mean) / sample SD). The HDL-c Z-score was multiplied by (–1) to indicate higher cardiovascular risk with increasing value. The composite index of blood lipids and fasting glycaemia was the sum of the four Z-scores. The mean of this continuously distributed metabolic composite index was therefore zero by definition [18]. For analysis of the differences between BMI (normal vs. overweight/obese) and MF/BF (tertiles: unfit, average, fit), an ANOVA model was used for continuous variables. ANCOVA (BMI x MF) was applied to determine differences in lipids, CMRSI and LMCRI and to test the effect of MF/BF where age and smoking were used as covariates. As a post hoc test, Bonferroni’s pairwise comparisons were applied in all analyses.

## Results

Table 1 shows the characteristics of all participants grouped by their BMI. Of 6,095 participants, 803 (13.1 %) were smokers. All study variables, except the MF, MF/BM and HDL-c, were higher in the overweight/obese group than in those with normal BMI (*p* < 0.001).

**Table 1:** Characteristics of the participants, anthropometrics, muscular fitness, clinical and metabolic biomarkers, LMCRI and CMRSI by BMI

Characteristics of the participants		BMI Groups	
	All participants (*n* = 6,095)	BMI Normal (*n* = 4,814)	BMI OW/OB (*n* = 1,281)
Age (years)	29.6 ± 11.7	25.1 ± 9.3	36.1 ± 11.8*
Smoking n, (%)	803 (13.1)	604 (12.5)	199 (15.5)
BM (kg)	62.3 ± 11.8	58.2 ± 8.3	76.1 ± 11.3*
Height (m)	1.65 ± 0.1	1.65 ± 0.09	1.66 ± 0.09*
BMI (kg•m^−1^)	22.8 ± 3.4	21.3 ± 2.0	27.6 ± 2.6*
WC (cm)	81.0 ± 11.8	75.3 ± 6.8	92.7 ± 11.4*
SBP (mmHg)	114.0 ± 14.0	112.7 ± 13.5	118.9 ± 14.7*
DBP (mmHg)	70.0 ± 10.0	69.3 ± 9.9	72.9 ± 10.0*
MBP (mmHg)	85.8 ± 9.6	85.8 ± 9.6	85.9 ± 9.6
MF (kg)	29.9 ± 10.3	29.4 ± 10.1	32.1 ± 11.0*
MF / BM (kg)	0.494 ± 0.189	0.508 ± 0.191	0.444 ± 0.175*
Total cholesterol (mg/dL)	165.4 ± 44.6	155.4 ± 38.4	185.8 ± 49.0*
Triglycerides (mg/dL)	114.9 ± 80.7	89.2 ± 45.4	168.1 ± 107.0*
LDL-c (mg/dL)	92.5 ± 34.4	86.7 ± 28.3	105.3 ± 42.9*
HDL-c (mg/dL)	47.7 ± 16.8	49.1 ± 18.3	44.4 ± 12.0*
Glucose (mg/dL)	80.3 ± 13.6	77.3 ± 12.2	86.1 ± 14.2*
LMCRI	0.167 ± 1.337	−0.291 ± 0.841	1.127 ± 1.644*
CMRSI	−1.064 ± 0.369	−1.102 ± 0.365	−0.899 ± 0.343*

Mean ± standard deviation. ANOVA test was used to examine any significant difference by BMI group

*OW/OB* overweight/obese; *BM* body mass; *BMI* body mass index; *WC* waist circumference; *SBP* systolic blood pressure; *DBP* diastolic blood pressure; *MBP* Mean blood pressure; *MF* muscular fitness; *LMCRI* lipid–metabolic cardiovascular risk index; *CMRSI* Cardiometabolic Risk Factor Score Index

* Differences between normal and overweight/obese BMI groups (*P* < 0.001)

Table 2 shows all of the study variables linked to the LMCRI and the CMRSI, analyzed according to BMI and MF/BM tertiles. Unfit–MF/BM/overweight-obese subjects (first tertile) had higher triglycerides, LDL-c, CMRSI and LMCRI and lower HDL cholesterol compared with fit–MF/BM/normal-weight participants. In the subgroup with average MF/BM (second tertile) fitness, the concentration of triglycerides and glucose fasting, were higher in the overweight-obese compared with the BM-normal subgroup. In addition, it can be observed that in all categories, high MF/BM indicated lower LMCRI (F: 142.2; *P* < 0.001) and CMRSI (F: 16.4; *P* < 0.001); this result was independent of BMI.

**Table 2:** Cardiometabolic Risk Score Risk Index and Lipid-Metabolic Cardiovascular Risk Index in BMI and MF/BM tertiles subgroups

MF/BM tertiles	Study variables	BMI Groups	
		BMI Normal	BMI OW/OB
Fit (third tertile)		(*n* = 1,758)	(*n* = 294)
	Total cholesterol (mg/dL)	142.9 ± 31.4	148.8 ± 36.4
	Triglycerides (mg/dL)	83.1 ± 38.6	100.4 ± 44.0*
	LDL-c mg/dL)	81.6 ± 26.3	82.6 ± 24.5
	HDL-c (mg/dL)	45.9 ± 12.4	43.5 ± 18.1
	Glucose (mg/dL)	70.6 ± 10.7	71.9 ± 8.6
	WC	74.9 ± 5.2	86.0 ± 6.0*
	MPB	87.0 ± 9.8	87.5 ± 10.5
	CMRSI	−0.423 ± 0.791	0.018 ± 0.965*
	LMCRI	−1.961 ± 0.340	−0.425 ± 0.371*
Average fit (second tertile)		(*n* = 1,576)	(*n* = 399)
	Total cholesterol (mg/dL)	156.3 ± 35.0	148.8 ± 23.9*
	Triglycerides (mg/dL)	88.2 ± 43.2	115.2 ± 64.1*
	LDL-c mg/dL)	88.6 ± 26.1	85.8 ± 24.2
	HDL-c (mg/dL)	50.5 ± 25.2	40.4 ± 10.9*
	Glucose (mg/dL)	74.9 ± 11.4	79.9 ± 11.1*
	WC	73.8 ± 6.4	87.1 ± 7.0*
	MPB	87.2 ± 9.6	91.9 ± 8.5*
	CMRSI	−0.363 ± 0.723	0.064 ± 0.878*
	LMCRI	0.859 ± 0.397	1.047 ± 0.325*
Unfit (first tertile)		(*n* = 1,480)	(*n* = 588)
	Total cholesterol (mg/dL)	159.9 ± 32.5	151.2 ± 29.3
	Triglycerides (mg/dL)	94.2 ± 42.4	119.4 ± 70.4*
	LDL-c mg/dL)	89.5 ± 26.6	92.6 ± 31.5
	HDL-c (mg/dL)	53.7 ± 14.4	38.5 ± 14.2*
	Glucose (mg/dL)	76.8 ± 11.5	80.5 ± 10.1*
	WC	72.7 ± 5.6	83.5 ± 8.6*
	MPB	86.3 ± 9.0	89.2 ± 9.1
	CMRSI	−0.355 ± 0.676	0.132 ± 1.104*
	LMCRI	0.941 ± 0.298	1.044 ± 0.351*

Age and smoking were used as covariates (ANCOVA). Differences within the respective BMI groups between the fitness tertiles were compared with the unfit subgroup using Bonferroni correction

*MF/BM* muscular fitness/body mass; *OW/OB* overweight/obese; *WC* waist circumference; *MBP* mean blood pressure; *LMCRI* lipid-metabolic cardiovascular risk index; *CMRSI* Cardiometabolic Risk Factor Score Index

* *P* < 0.001. Mean ± standard deviation

## Discussion

The results of the present research demonstrate the importance of the relationship between MF/BM and several health indices linked to cardiometabolic risk in Latin American adults. These results have greater relevance when considering that most studies of this type (with a large sample size) have focused on other population types [3, 7]. The present study therefore responds to the need to increase the amount of scientific evidence for cardiometabolic risk factors in different populations [2].

The reliable measurement of muscle strength in the different muscle groups takes a long time, but the handgrip strength test is considerably easier, faster, and a more cost-effective way of predicting total muscle strength, defined as MF [19]. In this sense, it is important to clarify that handgrip strength is not trainable, but that it has been related to changes in muscle strength associated with age, training status and nutritional health [2, 8, 14].

Excess total fat (>25 %) and abdominal obesity (waist circumferences >102 cm) in adult men has been shown to be inversely related to leg and arm MF [6], and the results of the present study agree with this. A low level of MF has been identified as a potential risk factor for several different cardiometabolic diseases [2, 6]. Recently, a 4-year study of 139,691 participants, the PURE study (Prospective Urban Rural Epidemiology Study) [14], showed that a reduction of 5 kg in grip strength estimated using a handgrip dynamometer was inversely related to all-cause mortality (hazard ratio 1.16; IC 95 % 1.13–1.20; *p* < 0.0001), cardiovascular mortality (hazard ratio 1.17; IC 95 % 1.11–1.24; *p* < 0.0001), and coronary ischemic events (hazard ratio 1.07; IC 95 % 1.02–1.11; *p* = 0.002).

Steene-Johannessen et al. [20] reported that in young people, independent of adiposity and cardiorespiratory fitness, higher MF was associated with lower levels of chronic inflammation markers, such as C reactive protein, leptin and TNF-α, which can promote systemic low-grade inflammation. It thus appears that an adequate ratio of MF/BM is of increased importance in individuals with a higher fat mass, particularly in those with a higher visceral fat mass. This is presumably due to the burden of chronic inflammation associated with these adipocytes [3].

The present study found that participants with a higher level of MF/BM had improved anthropometric characteristics, metabolic biomarkers and lower cardiometabolic risk (CMRSI and LMCRI), independent of their nutritional status (normal or overweight/obese). The use of CVD risk factors as continuous variables can provide more information about metabolic syndrome in adults. In our study, we used two cardiometabolic indexes that combined some of the biochemical parameters implicated in the metabolic syndrome, such as glucose, HDL-C, LDL-C and triglyceride concentrations. Several groups have recently used a continuous score [11, 18] and several authors have postulated that Z-scores are population specific [18]. In fact, information is not reduced when we use continuous variables and the composite Z-score can be used as a continuous or dichotomous variable. Two potential sources of bias in the calculation of the metabolic index should be noted: first, the mean triglyceride concentration is used in the calculation to standardize the values found: standardized value = [(observed value-mean)/standard deviation]; triglyceride concentrations show a non-Gaussian distribution in any population; secondly, other risk factors are not measured, such as adiponectin or insulin, however, the mean Z-score may still indicate a reasonably correct level of metabolic health. These results are in agreement with those published previously in the literature for populations who possess the same sociocultural characteristics, although with different age groups [11]. The positive effect of MF on health has also been observed in other population types, children and adolescents [3], adults [2] and the elderly [5]. Overall, these results demonstrate the relevance of including this marker as a part of the clinical assessment of patients [11].

On the other hand, differences in our study could be explained in part by differences in socio-economic status and the racial/ethnic composition of the populations and covariates included [14]. This is in contrast to countries such as the US, where race and muscular strength are closely linked [1]. Lower birth weight, which is associated with poorer childhood muscular fitness, is also more common in children from lower socioeconomic status families [1]. Results from the PURE study [14] showed MF values highest among those from Europe/North America, lowest among those from South Asia, South East Asia and Africa, and intermediate among those from China, South America, and the Middle East, however, higher performance in a number of measures of fitness have been observed in urban compared to rural Ecuadorian [21] and Chilean [22] youth, suggesting a positive association between the economic development of an area and fitness, similar to that reported in high income countries. Also, because MF is dependent on nutritional status and racial/ethnic composition [11], several studies normalized grip strength as strength per body mass [2, 3, 10]. The rapid adoption of a sedentary lifestyle in low to middle-income countries (LMIC) is a major public health concern, as chronic diseases such as diabetes are increasing at a more rapid rate in LMIC than high income countries. The prevalence of overweight/obese Colombian households increased from 2000 to 2014 (38.2 to 43.1 %) along with concomitant decreases in undernourished households (13.7–10.6 %, respectively) and households with the dual burden of overweight and underweight 2010 (3.5 and 5.1 %, households) [23].

MF has also been shown to have a positive influence on the body, independent of other powerful health markers, such as cardiorespiratory fitness [2, 3, 11, 13]. This independent influence may be because strength training promotes the synthesis of new proteins via an intracellular signaling pathway that is different from that of endurance training [24]. These differentiated signaling pathways demonstrate the importance of including MF training in physical exercise programs that aim to improve health, especially when considering that studies have demonstrated that even the level of physical activity is not as important a variable with regard to presenting a healthy lipid-metabolic profile compared to the level of MF and cardiorespiratory fitness [11, 13]. Similarly, in the Pan-European HELENA study, Artero et al. [12] found an inverse association between MF and inflammatory biomarkers in overweight/obese Spanish adolescents after adjusting for gender, age, cardiorespiratory fitness, and weight status; this relationship was marginally non-significant in children of normal weight. These findings suggest that overweight and obese adolescents may exhibit less adverse profiles if they maintain appropriate MF levels.

Other studies have shown that strength-training programs appear to be sufficient for achieving healthy cardiometabolic profiles in healthy and overweight subjects [25]. Unlike aerobic training, strength training appears able to have a protective effect on glycosylated hemoglobin glycaemia and lipid profiles, even after short-term interruptions to the training program [26].

Research has also shown that men and women with a low level of MF have a higher probability of gaining ~10 kg in weight, independent of BMI and cardiorespiratory fitness [27], however, in the case of the present study, the group of participants with an overweight/obese BMI and with the highest levels of MF/BM showed lower cardiometabolic risk than their peers from the first and second tertile (unfit and average, respectively).

It has been shown in the literature that even individuals with high levels of adiposity or BMI can have several different parameters linked to metabolic abnormalities and cardiovascular disease within normal ranges [28, 29]. A metabolically healthy obese person can thus have a series of indices that favor their health, such as a greater amount of type-II muscle fibers, high levels of insulin sensitivity, reduced muscle lipid infiltration, high metabolic activity linked to glucose storage and use, absence of hypertension, and favorable lipid, inflammation, hormonal, liver enzyme and immune profiles [3, 27, 28, 30]. Metabolically healthy individuals with obesity and sarcopenic obesity showing high levels of strength also perform better on indicators of health [31]. This again suggests encouraging the use of not only quantity but also quality of muscle mass as a marker of cardiometabolic health [3].

Many Latin American countries, including Colombia, have experienced significant economic changes, urbanization, and other factors that have drastically changed habits related to diet and physical activity, significantly affecting body composition and other health-related fitness measurements [23]. It is well known that cardiorespiratory fitness and MF [32] are better predictors of cardiovascular disease risk factors in young people than BMI, and both prospective and case-control studies have shown that even with a normal BMI, those with lower physical fitness are at an increased risk of cardiovascular disease and premature death [2, 33]. Obesity and physical inactivity are leading CVD risk factors among Hispanic/Latino adults, raising concerns about whether an increased risk from these conditions also manifests at younger ages [23]. These changes are contributing to a global increase in the prevalence of non-communicable diseases, and therefore, the inclusion of MF within health surveillance systems is justifiable and has been recommended by other authors [2–4, 11]. Describing the magnitude of these risk factors in adults is important for prioritizing prevention of disease and public health efforts.

There are some limitations to this study. The observations of our study are limited by the use of a descriptive and cross-sectional design, and therefore the direction of causality cannot be determined. Another limitation was that MF was measured by grip strength. Aspects of the protocol such as allowance for hand size and dominance, posture, joint position, effort and encouragement, frequency of testing and time of day, and the training of the assessor can influence the absolute values and precision of grip strength measurements. Grip strength performance was recorded as the best score from either hand, without consideration of hand dominance, however, since there is substantial covariance between strength capacity and body mass—and, moreover, the links between muscle strength and both physical function and chronic health are mediated by the proportion of strength relative to body mass—grip strength was normalized as strength per body mass [i.e. (grip strength in kg)/(body mass in kg)]. Future research is needed to better describe the age- and sex-specific trajectories of strength as a predictor of comorbidities across the lifespan and, perhaps just as importantly, to apply robust analyses that can compartmentalize risk into hierarchical categories. Finally, this study included the use of BMI as a measure of adiposity to define adult obesity and did not use expensive tools that are sometimes difficult to transport to the field (e.g., duel-energy X-ray absorptiometry, air displacement plethysmography, bioelectrical impedance); thus, although reference data is available, these are difficult measurements to obtain accurately and are not commonly used [34]. Nonetheless, BMI is widely recognized as an appropriate tool with which to screen obese adults and to define overweight status, as a state of excessive weight relative to height, regardless of body composition [35, 36]. Because BMI does not measure body composition and adiposity, it only is a screening tool to identify obesity, as defined by excessive adiposity [35].

## Conclusions

This study found an inverse association between high MF/BM values and two important cardiometabolic risk factor indicators (CMRSI and LMCRI) that are independent of BMI for a large sample of Latin American adults, however, individuals with high MF/BM and normal BMI had a better cardiometabolic protection profile. It is the opinion of the researchers that in the context of public health, these results underline the importance of promoting strength training in programs aiming improve people’s general health, while also increasing the amount of evidence supporting the inclusion of MF assessment with a handgrip strength test within clinical evaluations.

## Abbreviations

ANCOVA: Analysis of covariance
ANOVA: Analysis of variance
BMI: Body mass index
CMRSI: Cardiometabolic risk score index
HDL-c: High-density lipoprotein cholesterol
ISAK: International Society for the Advancement of Kinanthropometry
LDL-c: Low-density lipoprotein cholesterol
LMCRI: Lipid-metabolic cardiovascular risk index
LMIC: Low to middle-income countries
MBP: Mean blood pressure
MF: Muscular fitness
MF/BM: Strength relative to body mass
TEM: Technical error of measurement
WC: Waist circumference
